# The Involvement of PI3K-Mediated and L-VGCC-Gated Transient Ca^2+^ Influx in 17β-Estradiol-Mediated Protection of Retinal Cells from H_2_O_2_-Induced Apoptosis with Ca^2+^ Overload

**DOI:** 10.1371/journal.pone.0077218

**Published:** 2013-11-05

**Authors:** Yan Feng, Baoying Wang, Fangying Du, Hongbo Li, Shaolan Wang, Chenghu Hu, Chunhui Zhu, Xiaorui Yu

**Affiliations:** 1 Department of Genetics and Molecular Biology, School of Medicine, Xi’an Jiaotong University, Xi’an, China; 2 Key Laboratory of Environment-and-Gene Related Diseases of the Ministry of Education, School of Medicine, Xi’an Jiaotong University, Xi’an, China; Karolinska Institutet, Sweden

## Abstract

Intracellular calcium concentration ([Ca^2+^]_i_) plays an important role in regulating most cellular processes, including apoptosis and survival, but its alterations are different and complicated under diverse conditions. In this study, we focused on the [Ca^2+^]_i_ and its control mechanisms in process of hydrogen peroxide (H_2_O_2_)-induced apoptosis of primary cultured Sprague-Dawley (SD) rat retinal cells and 17β-estradiol (βE2) anti-apoptosis. Fluo-3AM was used as a Ca^2+^ indicator to detect [Ca^2+^]_i_ through fluorescence-activated cell sorting (FACS), cell viability was assayed using MTT assay, and apoptosis was marked by Hoechst 33342 and annexin V/Propidium Iodide staining. Besides, PI3K activity was detected by Western blotting. Results showed: a) 100 μM H_2_O_2_-induced retinal cell apoptosis occurred at 4 h after H_2_O_2_ stress and increased in a time-dependent manner, but [Ca^2+^]_i_ increased earlier at 2 h, sustained to 12 h, and then recovered at 24 h after H_2_O_2_ stress; b) 10 μM βE2 treatment for 0.5-24 hrs increased cell viability by transiently increasing [Ca^2+^]_i_, which appeared only at 0.5 h after βE2 application; c) increased [Ca^2+^]_i_ under 100 µM H_2_O_2_ treatment for 2 hrs or 10 µM βE2 treatment for 0.5 hrs was, at least partly, due to extracellular Ca^2+^ stores; d) importantly, the transiently increased [Ca^2+^]_i_ induced by 10 µM βE2 treatment for 0.5 hrs was mediated by the phosphatidylinositol-3-kinase (PI3K) and gated by the L-type voltage-gated Ca^2+^ channels (L-VGCC), but the increased [Ca^2+^]_i_ induced by 100 µM H_2_O_2_ treatment for 2 hrs was not affected; and e) pretreatment with 10 µM βE2 for 0.5 hrs effectively protected retinal cells from apoptosis induced by 100 µM H_2_O_2_, which was also associated with its transient [Ca^2+^]_i_ increase through L-VGCC and PI3K pathway. These findings will lead to better understanding of the mechanisms of βE2-mediated retinal protection and to exploration of the novel therapeutic strategies for retina degeneration.

## Introduction

Intracellular Ca^2+^ concentration ([Ca^2+^]_i_) plays a vital role in regulating many fundamental cellular processes, such as gene regulation, cell proliferation, cell survival, and apoptosis [1]. Ca^2+^ homeostasis is tightly regulated and the disturbances in Ca^2+^ homeostasis have been implicated in degenerative diseases such as Parkinson's disease (PD), Alzheimer’s disease (AD) and Huntington’s disease (HD) [2,3]. The increase of [Ca^2+^]_i_ is mediated by two closely related mechanisms: excessive release of Ca^2+^ from endoplasmic reticulum (ER) stores and store-operated Ca^2+^ entry (SOCE), the Ca^2+^ influx process through plasma membrane (PM) channels following the release of Ca^2+^ from the ER stores [4]. Specifically, [Ca^2+^]_i_ alterations are different under diverse conditions. Accumulating evidence suggests that both the excessive elevation of [Ca^2+^]_i_ and the loss of [Ca^2+^]_i_ are crucial for degenerative diseases [5]. Increased [Ca^2+^]_i_ leads to the inappropriate activation of Ca^2+^-dependent processes, which are normally inactive or operate at low Ca^2+^ levels, thus causing metabolic derangements that ultimately lead to cell death [6]. In contrast, chronic depletion of ER Ca^2+^ influences ER-dependent processes and also inhibits Ca^2+^-dependent cellular functions. Furthermore, loss of Ca^2+^ homeostasis leads to the ER stress response and apoptosis [7]. Alternatively, increased Ca^2+^ entry has been implicated in both cell survival and cell death processes, and Ca^2+^ has been shown to exert a biphasic effect on cellular growth. Furthermore, a modest increase in [Ca^2+^]_i_ promotes cell proliferation, whereas relatively high [Ca^2+^]_i_ leads to increased mitochondrial Ca^2+^ and accounts for the release of pro-apoptotic factors resulting in cell death [8,9]. Therefore, diverse Ca^2+^ actions in different cells must be dependent on the cellular concentration as well as the locations [8].

Oxidative stress-induced cell apoptosis has been implicated in various diseases such as degeneration of nervous system [10]. Hydrogen peroxide (H_2_O_2_) has been implicated in triggering apoptosis in various cell types and has become a well-established in vitro model for studying the pathology of oxidative stress in central nervous system (CNS) disorders [11]. The retina is a part of CNS [12]. Apoptosis has been described in many retinal degenerative diseases such as retinitis pigmentosa (RP) and age-related macular degeneration (AMD) [13]. Many studies have focused on [Ca^2+^]_i_ increases in degenerative disorders of CNS [14,15]; however, the effects of [Ca^2+^]_i_ reduction and deficiency have also been studied and shown to play a role in degenerative disorders of CNS [16]. These different results may be caused by temporal and spatial specificity. For example, an early increase and subsequent decline in [Ca^2+^]_i_ may occur or Ca^2+^ may be reduced in specific cellular compartments and increased in other compartments [17]. 

Estrogen is an antioxidant that exerts various role by itself or by regulating intracellular signaling pathways [18], and it has also been established that estrogen plays a role in Ca^2+^ homeostasis [19]. Nevertheless, the reports regarding the effects of estrogen on Ca^2+^ homeostasis in nervous system protection are inconsistent. Several studies showed that estrogen exerts neuroprotection by increasing [Ca^2+^]_i_ [20–22], but other studies showed that the same result occurred via [Ca^2+^]_i_ reduction [23,24]. These apparently conﬂicting results may be due to the differences in the study models, the intensity of injury or the timing of the [Ca^2+^]_i_ assessment. 

Several recent reports have shown that both estrogen receptor (ER) subtypes, ERα and ERβ, are present in the retina [25,26]. Evidence suggests that estrogen most likely plays a direct role in regulating the physiological processes of the retina [27]. Furthermore, 17β-estradiol (βE2), an extremely potent bioactive estrogen, attenuated the H_2_O_2_-induced apoptosis of retinal cells in vitro and inhibited light-induced photoreceptor apoptosis in vivo, suggesting that βE2 has retinal protective properties [28,29]. However, the roles of [Ca^2+^]_i_ in apoptosis and anti-apoptosis in our study model remain unknown. In this study, we detected the [Ca^2+^]_i_ of primary cultured Sprague-Dawley (SD) rat retinal cells treated with different concentrations of H_2_O_2_ or βE2 and at different time points after H_2_O_2_ or βE2 treatment. Next, we measured [Ca^2+^]_i_ under βE2 and H_2_O_2_ co-treatment, and we explored the controlling mechanisms of [Ca^2+^]_i_. Consequently, we found that treatment with 100 μM H_2_O_2_ led to primary cultured SD rat retinal cell injury and apoptosis, while treatment with 10 μM βE2 played a protective role. Both completely different roles were mediated by increasing the [Ca^2+^]_i_, which occurred at the early stage of apoptosis and at 0.5 h after βE2 treatment. Furthermore, both of the increased [Ca^2+^]_i_ under completely opposite conditions were partially due to extracellular [Ca^2+^]_i_. Importantly, the transient [Ca^2+^]_i_ increase induced by βE2 was gated by the L-type voltage-gated Ca^2+^ channels (L-VGCC) and phosphatidylinositol-3-kinase (PI3K) was involved, but it was not involved in the H_2_O_2_-induced [Ca^2+^]_i_ increase.

## Materials and Methods

### 2.1: Animals and Chemicals

SD rats (obtained on postnatal days 0-3, body weights of 5-12 g) were housed in a controlled environment in a specific pathogen-free animal center. The temperature was maintained at 24±2°C, the humidity was 52±10% and fresh air was circulated continuously. All of the procedures used in the experiments were approved by the Institutional Animal Ethics Committee, Medical School of Xi’an Jiaotong University (permission No. 2009-12) and conformed to accepted ethical standards of the Animals in Research and the Association for Research in Vision and Ophthalmology statement for the use of animals in vision and ophthalmic research. 

H_2_O_2_ was purchased from Xi’an Pure Chemical Industries (Xi’an, Shaanxi, China). Fetal Bovine Serum (FBS) and phenol red free 1:1 DMEM/F-12 were obtained from Hyclone (Logan, Utah, USA). Poly-lysine, βE2, Hoechst 333342 dye and nifedipine, an L-VGCC blocker, were purchased from Sigma (St. Louis, Missouri, USA). We used 95% ethanol as the solvent to make the βE2 stock solution at a concentration of 1x10^-2^ M. Fluo-3 AM, an indicator of intracellular Ca^2+^ levels, was purchased from Biotium (Hayward, Calif., USA). We used Dimethylsulfoxide (DMSO) as the solvent for making 5 mM Fluo-3 AM stock solution and 20% Pluronic F-127 (5900) (offered by Biotium) in DMSO to facilitate AM ester solubilization. Trypsin, DMSO, 3-(4,5-dimethylthiazol-2-yl)-2, 5-diphenyltetrazolium bromide (MTT) and ethylene glycol tetraacetic acid (EGTA), an extracellular Ca^2+^ chelator, were purchased from Amresco (Solon, Ohio, USA). LY294002, a PI3K inhibitor, was purchased from Cayman (Ann Arbor, MI, USA). The Annexin V-FITC Apoptosis Assay Kit and bicinchoninic acid (BCA) Protein Assay Kit were purchased from Zhuhai Joincare Bioscience Ltd (Zhuhai, Guangdong, China), and radio immunoprecipitation assay (RIPA) buffer was purchased from Biotech (Biotechnology, Inc. of China). Anti-p-Akt and anti-Akt antibodies were purchased from Cell Signaling (Boston, Massachusetts, USA), and Anti-β-actin antibody was purchased from Santa Cruz Biotechnology (Santa Cruz, Calif., USA).

### 2.2: Primary Retinal Cells Cultures

We cultured primary retinal cells referencing other’s study [28] and making some revision. Neonatal SD rats were sacrificed (10–12 rats were needed for each 24-well or 6-well culture plate) and then the eyeballs were enucleated and immediately placed into a beaker containing D-Hanks solution. The retinas were removed from the pigment epithelium layer with the aid of a dissecting microscope under sterile conditions and were placed into a glass tube containing 1:1 Ham’s F-12-DMEM medium. The beaker containing the eyeballs and the tube containing the retinas were placed onto ice. The retina fragments were treated with 0.25% trypsin at 37°C for 8 mins and the digestion was terminated by adding three times the volume of 1:1 Ham’s F-12-DMEM containing 10% FBS. The suspension was filtered with a 200-mesh screen and centrifuged at 1000 rpm for 10 mins. After the supernatant was discarded, the cells were suspended, diluted with medium containing 10% FBS to 1x10^6^ cells/ml and plated onto 24-well or 6-well plates (Corning Costar) with 1 ml or 3 ml of cell suspension per well. Before culturing, all the plates were coated with poly-lysine (0.1 mg/ml) and maintained in a humid incubator overnight. Next, we washed the plates three times with sterile double distilled water (ddH_2_O), once with D-Hanks balanced salt solution, and then with 200 μl of medium, which provided a pre-environment for cell growth. The cells were cultured at 37°C in a 5% CO_2_ atmosphere until they were used at 4-6 days in vitro, during which the medium was replaced according to the cell growth and metabolism conditions.

### 2.3: Drug Treatment

After the cultures were maintained for 4-6 days in vitro, H_2_O_2_ and/or βE2 were added by bath application. Overall, 1 M H_2_O_2_ was prepared from 30% H_2_O_2_ dissolved in sterile cool PBS and was diluted with the medium to 10 mM. Next, the 10 mM H_2_O_2_ was diluted with the essential medium gradually to 200-25 μM, and 0 μM was regarded as the control. The 0.5-100 μM βE2 was prepared from the 1x10^-2^ M βE2 stock solution with the medium and was added to the cultures. We considered 0 μM as the control. The βE2 stock solution was dissolved in 95% ethanol, and a small amount of ethanol was present in the medium (<1%), but it had no effect on the primary cultured SD rat retinal cells [28]. Except for analyzing the time and dose dependency of H_2_O_2_ or βE2, we used H_2_O_2_ at a final concentration of 100 μM for 2 hrs/24 hrs and βE2 at a final concentration of 10 μM for 0.5 hrs to perform the experiments. To discover the source of increased [Ca^2+^]_i_, different concentrations of EGTA were added directly to the medium 1 hr before the application of 100 μM H_2_O_2_ for 2 hrs or 10 μM βE2 for 0.5 hrs to chelate the extracellular Ca^2+^. Under the co-application, we pre-treated cells with 10 μM βE2 treatment for 0.5 hrs before the application of 100 μM H_2_O_2_ for 2 hrs. To conduct the channel experiments and the mechanism study, the cultures were pre-conditioned for 2 hrs by nifedipine or for 0.5 hrs by LY294002 before the other treatments.

### 2.4 Cell Viability Assay

 To determine the cell viability of the primary cultured SD rat retinal cells, we performed an MTT assay. MTT was applied to the cultures at a final concentration of 0.5 mg/ml for 4 hrs at 37°C in 5% CO_2_, and the wells with no cells were used as blank controls. The medium was then removed, and DMSO was added to solubilize the colored formazan crystal product. The absorbance was determined at 490 nm on a Measurement Photometric multi-well plate reader (Electron Corporation Multiskan Spectrum, Thermo, Finland) with Skanlt RE for Mass 2.2 software after the plates were agitated at 37°C for 10 mins. All absorbance values were subtracted by the blank value, and the untreated cultures were considered as the control group. The mean cell viability for each condition was determined by averaging at least quadruplicate values, the fold change relative to the control was calculated, and the control values were normalized to 1. All experiments were performed using 3-5 separate experiments to confirm reproducibility.

### 2.5: Assessment of Apoptosis

After exposure to 100 μM H_2_O_2_ for 0-24 hrs, apoptosis was assayed by Annexin V/Propidium Iodide (PI) staining and Hoechst 33342 staining. For Annexin V/PI staining, the cells were collected, centrifuged at 1000 rpm for 5 mins, suspended and diluted with 1×binding buffer (Annexin V-FITC Apoptosis Assay Kit) to 5×10^5^ cells/ml. The 500 μl suspension was loaded with 5 μl Annexin V-FIFC and 10 μl PI for 15 mins. After incubated in the dark at room temperature, the cells were analyzed within one hour with a ﬂow cytometer (San Jose, California, USA). For Hoechst 33342 staining, 40 μl of suspension was dropped onto the slide, ﬁxed in 4% paraformaldehyde in PBS at room temperature for 20 mins and stained with 2 μg/ml Hoechst 33342 dye in the dark for 10 mins. The samples were then observed under a ﬂuorescence microscope (Nikon, Eclipse Ti, Japan) with ﬂuorescence excitation at 340 nm and emission at 510 nm. The cells with condensed DNA were counted as apoptotic cells, and the average apoptotic cells of each field were calculated. The sample fields with approximately 100 cells were randomly selected, and each sample was evaluated. The cells in 3-5 random fields/cultures were scored, and the counts were based on at least four separate cultures in each treatment condition.

### 2.6: Intracellular Ca^2+^ Measurement

 [Ca^2+^]_i_ detection was performed by FACS analysis [30]. After washing twice with PBS, the adherent cells were digested from plates with 300 μl 0.25% trypsin per well, and the digestion reaction was quenched by the addition of Ca^2+^-free medium containing 900 μl 10% FBS per well. The suspensions were collected and centrifuged at 1000 rpm for 10 mins. After discarding the supernatant, we suspended the cells with Ca^2+^-free PBS and incubated it in dark with 2 μM Fluo-3AM (Molecular Probes, Biotium) at 37°C for 30 mins and at room temperature for 15 mins. The sample without Fluo-3AM was considered as the blank control, whose fluorescence was represented as F_0_. Before detection, we washed the cells twice with PBS to minimize background fluorescence and nonspecific staining. The ﬂuorescence was measured at FL-1 (526 nm) in a ﬂow cytometer (Becton Dickinson, FACSCalibur-E4121, Becton Dickinson Immunocytometry systems driven by 2350 Qume, San Jose, California, USA) with an excitation laser at 488 nm, and at least 10,000 events per sample were acquired. The obtained image data were analyzed with Cell Quest Version 3.3 software and the Geo Mean of fluorescence (F) was used because its standard normal distribution was better compared to the mean fluorescence. All F values were subtracted by F_0_ to eliminate the background fluorescence and nonspecific staining. The relative F values of each treated group were expressed as the fold of control, with the F values of the control group normalized to 1. The changes of relative F values of Fluo-3AM represented the [Ca^2+^]_i_ alteration. To confirm the reproducibility, all experiments were performed at least 3-5 times with separate cultures. 

### 2.7: Western Blot Analysis

 The primary cultured retinal cells lysates were made by mixing cold RIPA buffer at a pH of 7.0 (the RIPA buffer consists of 20 mM Tris/HCl, 2 mM ethyleneglycoltetraacetic acid, 25 mM 2-glycerophosphate, 1% Triton X-100, 2 mM dithiothreitol, 1 mM vanadate, 1 mM phenylmethylsulfonyl fluoride and 1% aprotinin) with a 1 mM solution of the serine protease inhibitor phenylmethanesulfonyl fluoride (PMSF) (Sigma-Aldrich, St. Louis, MO) and a 10% solution of phosphatase inhibitor mixture P1260 (Applygen Technologies Inc., Beijing, China). The mixture was then homogenized on ice for 5 mins and centrifuged at 12000 g at 4°C for 20 mins. The BCA protein assay reagents (Pierce, Rockford, USA) were used to assess the concentration of the cell lysates. The assays were performed in triplicate, and the cell lysates were subsequently loaded onto a 12% sodium dodecyl sulfate (SDS) polyacrylamide gel, underwent electrophoresis and were subsequently transferred to a nitrocellulose membrane (Millipore, Bedford, MA) that was blocked with 5% non-fat dry milk in Tris-buffered saline (TBS, pH7.4) and incubated with anti-p-Akt and anti-Akt (1:1000, Cell signaling, Boston, USA) at 4°C overnight. After washing the membrane with TBS/T (TBS with 0.1%Tween 20), we applied goat anti-rabbit IgG (1:5000) labeled with horseradish peroxidase (HRP) at room temperature for 4 hrs, and then washed the membrane with TBS. Anti-β-actin antibody (1:2000, Santa Cruz Biotechnology, Santa Cruz, CA, USA) was used to verify the protein concentration. The ECL system (Thermo, USA) was used to visualize the protein bands. 

### 2.8: Statistical Analysis

All results were based on 3-5 independent replications with 4-6 samples per condition per experiment. Values shown in this study were expressed as the mean ±SD. Data were analyzed using the T-test for independent samples, or One-way ANOVA and the LSD post hoc test were used for multiple comparisons. P<0.05 was considered statistically significant for all tests. 

## Results

### 3.1: H_2_O_2_ induced the apoptosis of primary cultured SD rat retinal cells, and the [Ca^2+^]_*i*_ increased during the early apoptosis

**Figure 1:**
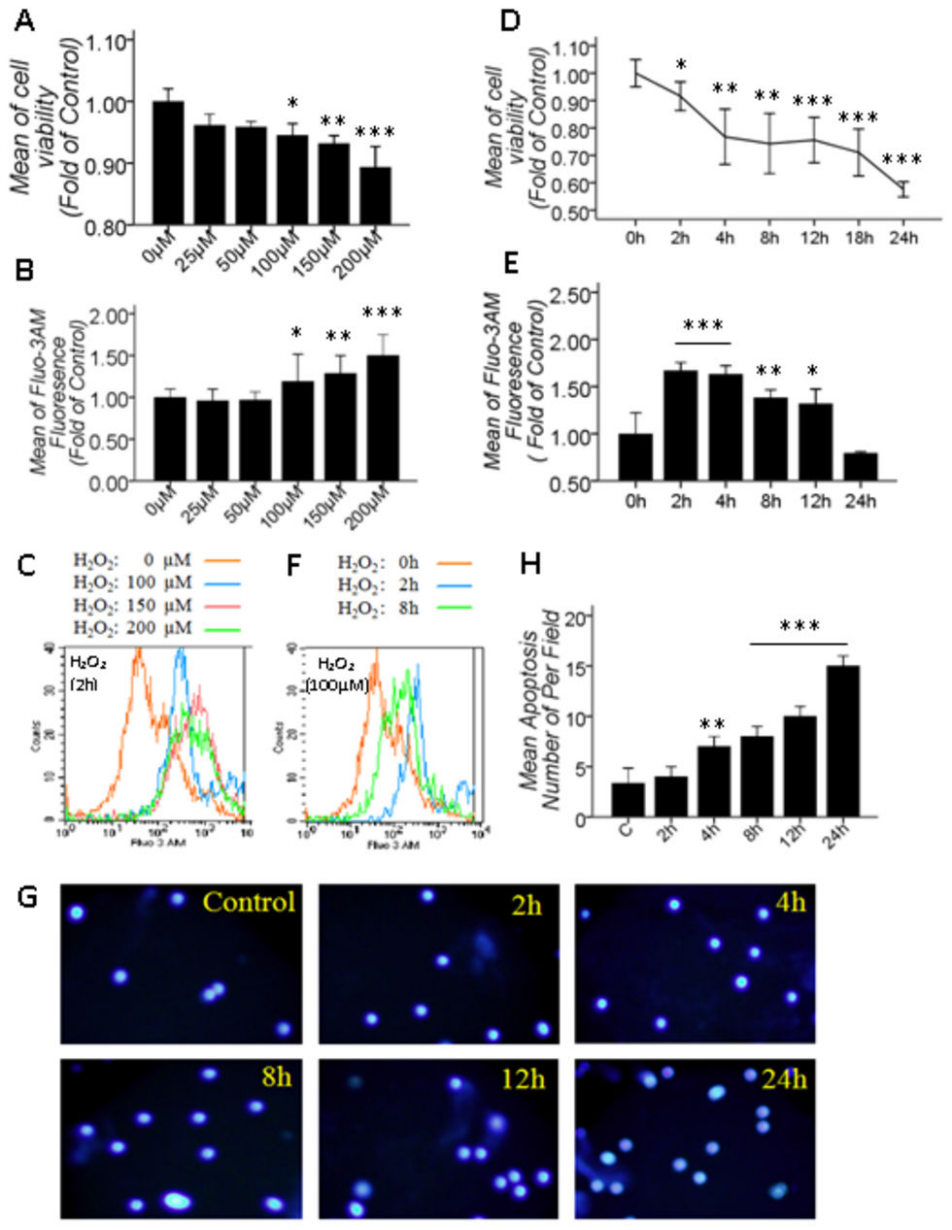
100 μM H_2_O_2_ induced primary cultured SD rat retinal cell apoptosis, which was associated with an increase in [Ca^2+^]_i_ at the early stage of apoptosis. A, B: Quantitative data of cell viability and [Ca^2+^]_i_ under different concentrations of H_2_O_2_ treatments for 2 hrs; D, E: Cell viability and [Ca^2+^]_i_ quantitative data at different time points after 100 μM H_2_O_2_-induced stress; C, F: The overlay figure of the representative statistical significance for B and E; G: Apoptosis assay using Hoechst 33342 staining at different time points after 100 μM H_2_O_2_-induced stress; H: Quantitative data of G. Values shown are the Mean ±SD. *represents P<0.05, **represents P<0.01 and ***represents P<0.001 compared with the control group by one-way ANOVA statistical analysis. (A, D, H: n indicates 3 independent replicates with 4 samples per condition per experiment; B, E: n indicates 3 independent replicates with 5 samples per condition per experiment.).

Most cell culture models of oxidative stress employ H_2_O_2_ as the pro-oxidant to induce oxidative stress because it is capable of altering the intracellular redox state of a cell and causing oxidative damage by its conversion to the highly reactive hydroxyl radical OH [28,31,32]. Furthermore, 100 µM H_2_O_2_ treatment for 24 hrs induced retinal cell apoptosis [28]. To ascertain the role of [Ca^2+^]_i_ in our study model and to dynamically observe the [Ca^2+^]_i_ alteration during apoptosis under a modest treatment condition, we performed the following experiments. First, cell viability and the [Ca^2+^]_i_ were assayed simultaneously at 2 h after treatment with different concentrations of H_2_O_2_. As shown in Figure 1, 25-200 µM H_2_O_2_ decreased cell viability (Figure 1A) but increased [Ca^2+^]_i_ in a dose-dependent manner (Figure 1B, C), which was significant at 100-200 µM. This finding indicated that 2 hrs after the application, 100-200 µM H_2_O_2_ reduced cell viability and caused Ca^2+^ overload. Next and importantly, we used 100 µM as the H_2_O_2_ concentration to dynamically and continuously observe apoptosis by Hoechst 33342 staining and [Ca^2+^]_i_ alteration during apoptosis, and cell viability was also assayed. The results showed that apoptosis was significant at 4 h, the significance increased over time (Figure 1G, H); however, the [Ca^2+^]_i_ increased remarkably at 2 h and 4 h, and this increase remained until 12 h but then gradually recovered to the control level at 24 h (Figure 1E, F). Cell viability was reduced in a time-dependent manner from 0 to 24 hrs (Figure 1D). Compared with control group, the 100 µM H_2_O_2_ treatment for 2 hrs caused a dramatic increase in [Ca^2+^]_i_ (P<0.001) and a slight decrease in cell viability; however, the 100 µM H_2_O_2_ treatment for 24 hrs caused a remarkable decrease in cell viability (P<0.001), but no significant alteration was discovered in [Ca^2+^]_i_ (Figure 1D, E), suggesting that the [Ca^2+^]_i_ increase occurs at the early stage of H_2_O_2_ induced apoptosis when cell injury is minimal. 

### 3.2: βE2 increased cell viability and protected primary cultured SD rat retinal cells from H_2_O_2_ injury, and the transient [Ca^2+^]_i_ increase was found to be involved in protection

**Figure 2:**
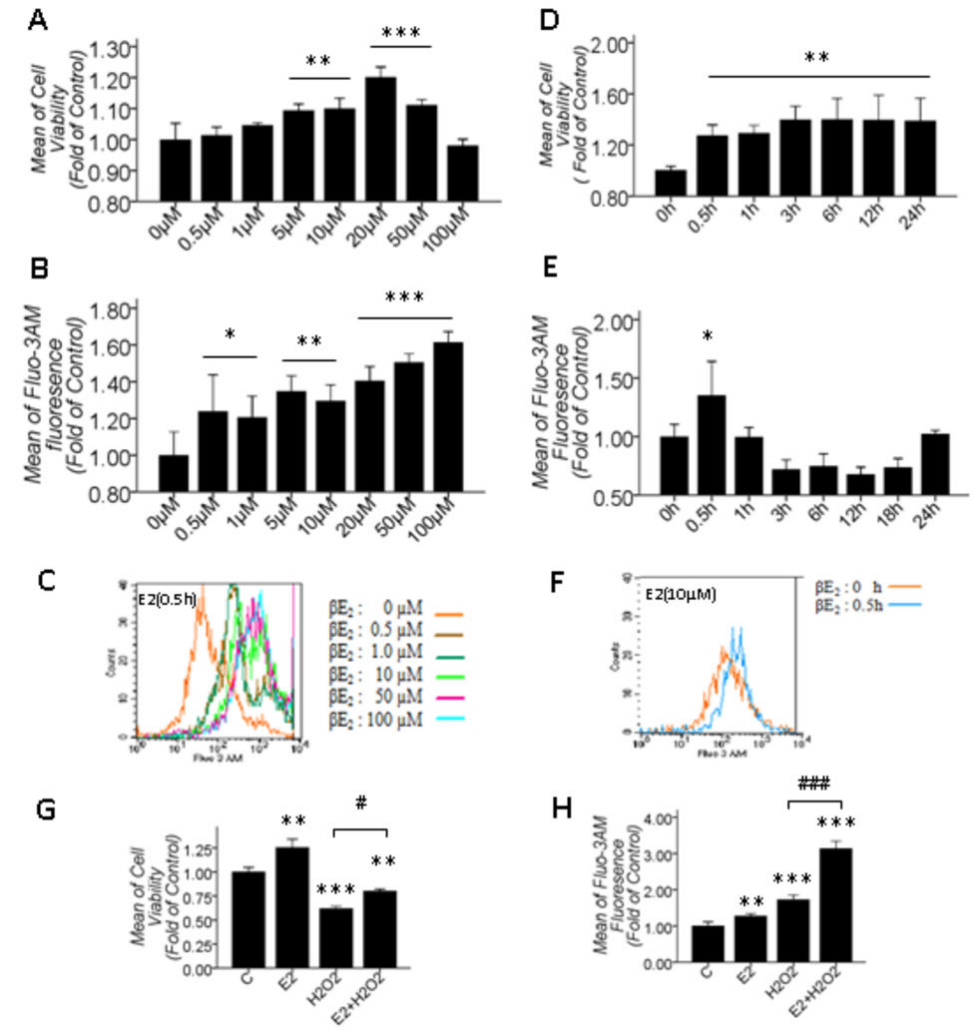
10 μM βE2 pretreatment for 0.5 hrs played a protective role in primary cultured SD rat retinal cells, which was associated with a transient and rapid increase in [Ca^2+^]_i_. A, B: Cell viability and [Ca^2+^]_i_ quantitative data under different βE2 concentrations for 0.5 hrs; D, E: Cell viability and [Ca^2+^]_i_ quantitative data at different time points after 10 μΜ βE2 treatment; C, F: The overlay figure of representative statistical significance for B and E; G, H: Cell viability and [Ca^2+^]_i_ quantitative data after 10 μM βE2 pretreatment for 0.5 hrs and 100 μM H_2_O_2_ treatment for 2 hrs. Values shown are the Mean ±SD. *represents P<0.05, **represents P<0.01 and ***represents P<0.001 compared with the control group; # represents P<0.05 and ### represents P<0.001 compared with the H_2_O_2_ application group by one-way ANOVA statistical analysis. (A, D: n indicates 3 independent replicates with 4 samples per condition per experiment; B, E: n indicates 3 independent replicates with 5 samples per condition per experiment; G, H: n indicates 3 independent replicates with 6 samples per condition per experiment.).

Pretreatment with 10 µM βE2 for 0.5 hrs effectively protected retinal cells from 100 µM H_2_O_2_-induced apoptosis [28]. To confirm whether or not [Ca^2+^]_i_ was involved in βE2-mediated protection in our model, we first observed the effects of different concentrations of βE2 treatment for 0.5 hrs and 10 µM βE2 treatment for different periods on cell viability and [Ca^2+^]_i_, respectively. The results showed that a range of 0.5-100 µM βE2 treatment for 0.5 hrs significantly increased [Ca^2+^]_i_ in a dose-dependent manner (Figure 2B, C), and 5-50 µM βE2 significantly increased cell viability (Figure 2A). However, at lower (0.5 and 1 µM) or higher (100 µM) concentrations of βE2, the treatment only increased [Ca^2+^]_i_ but had no effect on cell viability, which may be due to the concentration selectivity or because lower concentrations (0.5 and 1 µM) of βE2 are insufficient to increase cell viability and higher concentrations (100 µM) of βE2 are toxic for retinal cells. Interestingly, cell viability was significantly increased at 0.5-24 h after the application of 10 μM βE2 (Figure 2D), but the [Ca^2+^]_i_ increased significantly and rapidly only at 0.5 h after 10 μM βE2 treatment, fluctuated near the control level at 1-18 h, and then restored to the control level at 24 h (Figure 2E, F). Furthermore, under 10 μM βE2 pretreatment for 0.5 hrs and then 100 μM H_2_O_2_ treatment for 2 hrs, 10 μM βE2 pretreatment for 0.5 hrs significantly restored the decreased cell viability but significantly sharpened the increased [Ca^2+^]_i_ induced by 100 μM H_2_O_2_ for 2 hrs (Figure 2G,H), suggesting that βE2 increased cell viability and protected primary cultured SD rat retinal cells from H_2_O_2_ injury that is associated with immediate and transient [Ca^2+^]_i_ increases.

### 3.3: Both increased [Ca^2+^]_i_ induced by 100 μM H_2_O_2_ treatment for 2 hrs and 10 μM βE2 treatment for 0.5 hrs were caused by extracellular Ca^2+^ influx

**Figure 3:**
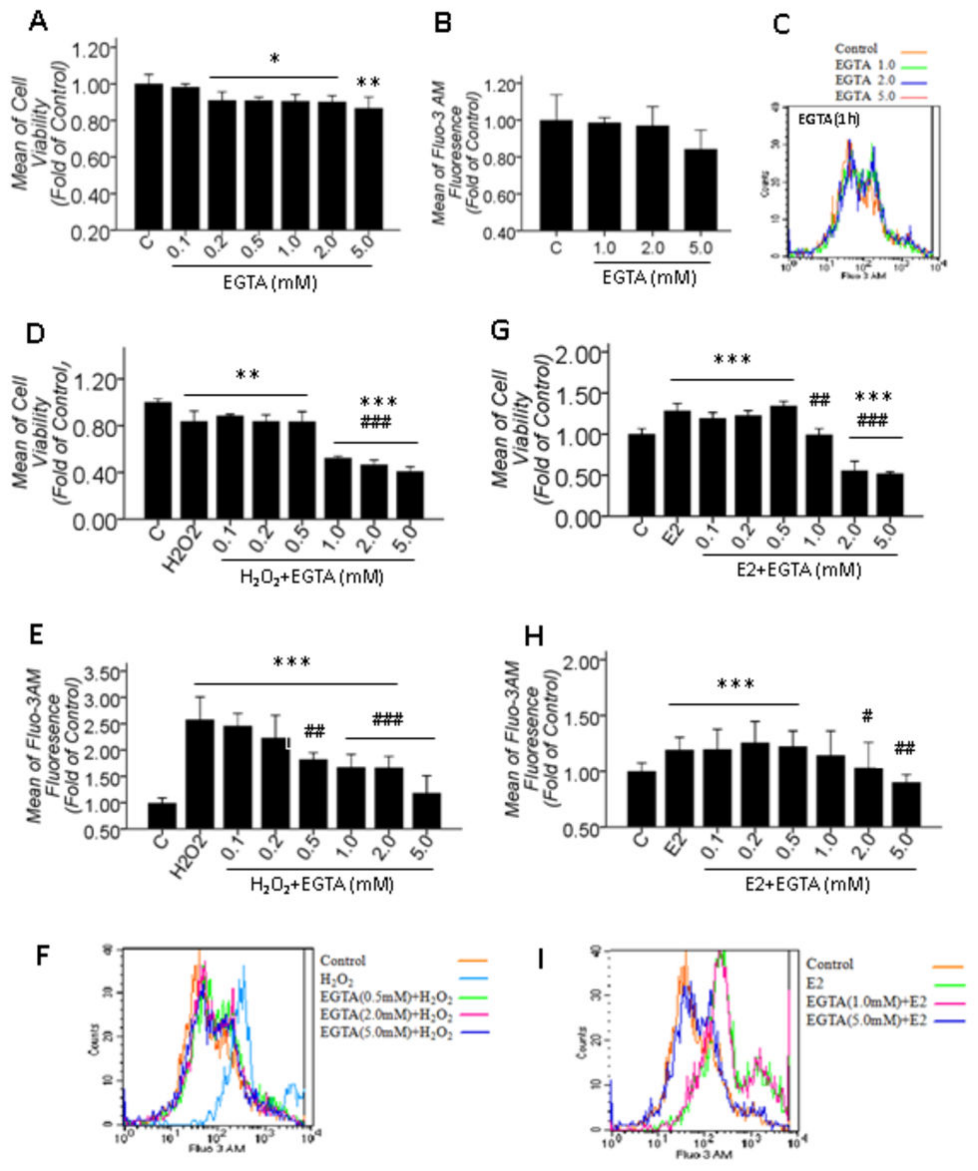
Sources of increased [Ca^2+^]_i_ induced by 100 μM H_2_O_2_ treatment for 2 hrs and 10 μM βE2 treatment for 0.5 hrs. A, B: The effects of different concentrations of EGTA treatment for 24 hrs on cell viability and EGTA treatment for 1 hr on [Ca^2+^]_i_; C: The overlay figure for B; D-F and G-I: The effect of different concentrations of EGTA pretreatment for 1 hr before H_2_O_2_ or βE2 application on the alteration of cell viability and [Ca^2+^]_i_ induced by H_2_O_2_ (D-F) or βE2 (G-I); F and I: The representative overlay figure for E and H. Values shown are the Mean ±SD. *represents P<0.05, **represents P<0.01 and ***represents P<0.001 compared with the control group; # represents P<0.05, ## represents P<0.01 and ### represents P<0.001 compared with the H_2_O_2_ or βE2 application groups by one-way ANOVA statistical analysis. (A, D, E: n indicates 4 independent replicates with 5 samples per condition per experiment; B, G, H: n indicates 4 independent replicates with 6 samples per condition per experiment.).

Ca^2+^ homeostasis is strictly controlled by channels, pumps and exchangers functioning as gates for Ca^2+^ entry and release. A cell becomes activated because of an external signal, which results in up to an 100-fold increase in the [Ca^2+^]_i_ caused by the uptake of extracellular Ca^2+^ and/or the release of intracellular Ca^2+^ stores. To confirm whether the increased [Ca^2+^]_i_ in our model treated with 100 μM H_2_O_2_ for 2 hrs or 10 μM βE2 for 0.5 hrs is due to the extracellular Ca^2+^ influx, we preliminarily detected the [Ca^2+^]_i_ before and after adding EGTA, a chelator of extracellular Ca^2+^, in the presence and absence of H_2_O_2_ or βE2, respectively. Simultaneously, cell viability was assayed. As shown in Figure 3, 0.2-5 mM EGTA treatment for 24 hrs decreased cell viability (Figure 3A), treatment with 1-5 mM EGTA for 1 hr had no effect on the [Ca^2+^]_i_ (Figure 3B, C). However, the effect of EGTA on the [Ca^2+^]_i_ was different in the presence of H_2_O_2_ or βE2. Based on previous experiments, we selected to pretreat the cells with 0.1-5 mM EGTA for 1 hr to chelate the extracellular Ca^2+^ before H_2_O_2_ or βE2 treatment. The results showed that 1-5 mM EGTA significantly aggravated the decrease in cell viability (Figure 3D), but 0.5-5 mM EGTA significantly attenuated the increase in [Ca^2+^]_i_ caused by the 100 μM H_2_O_2_-induced injury for 2 hrs (Figure 3E, F). This aggravating or attenuating effect was dose-dependent. Furthermore, 1-5 mM EGTA dose-dependently attenuated the increased cell viability and the increased [Ca^2+^]_i_ caused by 10 μM βE2 treatment for 0.5 hrs (Figure 3G, H, I). The attenuating impact of EGTA on the increased [Ca^2+^]_i_ induced by H_2_O_2_ or βE2 implicated that [Ca^2+^]_i_ increases under the two conditions were, at least, caused by extracellular sources. In this experiment, we monitored the pH before and after EGTA application and found that the low dose of EGTA did not alter the pH value of the medium, eliminating the effect of a change in pH as the cause of the increase in [Ca^2+^]_i_. 

### 3.4: L-VGCC mediated the [Ca^2+^]_i_ increase induced by 10 μM βE2 treatment for 0.5 hrs but did not mediate the [Ca^2+^]_i_ increase induced by 100 μM H_2_O_2_ for 2 hrs

**Figure 4:**
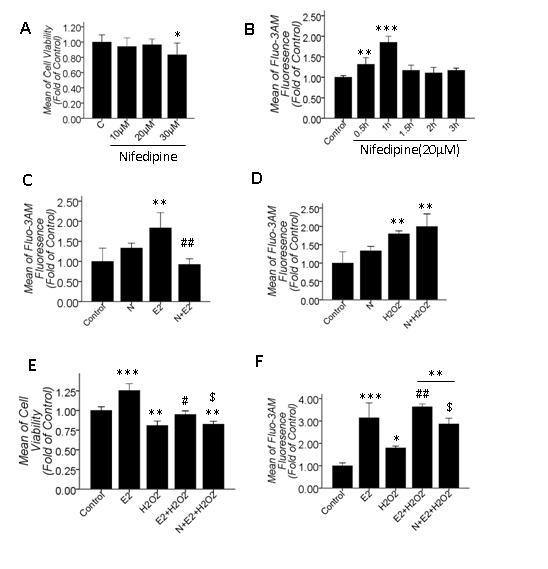
The effect of the L-VGCC blocker nifedipine (N) on the alteration of [Ca^2+^]_i_ during H_2_O_2_ injury and βE2 retinal protection. A: Cell viability under 10-30 μM nifedipine treatments for 24 hrs; B: [Ca^2+^]_i_ at different time points after 20 μM nifedipine application; C, D: The effects of 20 μM nifedipine pretreatment for 2 hrs on the increase in [Ca^2+^]_i_ due to 10 μM βE2 treatment for 0.5 hrs or 100 μM H_2_O_2_ treatment for 2 hrs; E, F: The attenuated effect of 20 μM nifedipine pretreatment for 2 hrs on the increased cell viability and [Ca^2+^]_i_ due to βE2 and H_2_O_2_ co-treatment. N is 20 μM nifedipine in B, C, D, E, and F. Values shown are the Mean ±SD. *represents P<0.05, **represents P<0.01 and ***represents P<0.001 compared with the control group; # represents P<0.05, ## represents P<0.01 compared with the H_2_O_2_ group (E, F) and ### represents P<0.001 compared with the βE2 group (C); $ represents P<0.05 compared with the βE2 and H_2_O_2_ co-application group by one-way ANOVA statistical analysis. (A: n indicates 5 independent replicates with 5 samples per condition per experiment; B, C, D, E, F: n indicates 3 independent replicates with 4 samples per condition per experiment.).

It has been suggested that estrogen potentiates L-VGCC in other cells [20–22]; however, it remained unknown whether L-VGCC gated the extracellular Ca^2+^ influx caused by 10 μM βE2 treatment for 0.5 hrs or 100 μM H_2_O_2_ treatment for 2 hrs in our model. To this end, we conducted several experiments using the L-VGCC blocker nifedipine. First, we measured the effect of nifedipine on the cell viability and found that treatment for 24 hrs with 10 μM and 20 μM nifedipine showed no effect on the cell viability, but 30 μM nifedipine significantly decreased the cell viability (Figure 4A). Second, we measured the [Ca^2+^]_i_ at different time points after 20 μM nifedipine treatment and found that the [Ca^2+^]_i_ increased at 0.5-1 h after 20 μM nifedipine application but later recovered (Figure 4B). When specifically blocking L-VGCC, the reactively impermanent increase in [Ca^2+^]_i_ occurred at 0.5-1 h after 20 μM nifedipine application because of the Ca^2+^ homeostasis. Afterwards, the [Ca^2+^]_i_ recovered to the resting level, and nifedipine began to develop its stable and innate effect. Third, we detected the blocking effect of nifedipine on increased [Ca^2+^]_i_ under two conditions and found that 20 μM nifedipine pretreatment for 2 hrs significantly attenuated the increased [Ca^2+^]_i_ induced by 10 μM βE2 treatment for 0.5 hrs (Figure 4C) but did not attenuate the increased [Ca^2+^]_i_ induced by 100 μM H_2_O_2_ treatment for 2 hrs (Figure 4D). L-VGCC gated the transient [Ca^2+^]_i_ increase induced by βE2 but did not gate the H_2_O_2_-induced [Ca^2+^]_i_ increase. Fourth, we analyzed the impact of nifedipine on βE2-mediated retinal protection and discovered that 20 μM nifedipine pretreatment for 2 hrs significantly attenuated βE2 protection against H_2_O_2_ injury (P=0.029, Figure 4E) and also significantly attenuated the increased [Ca^2+^]_i_ induced by βE2 and H_2_O_2_ co-treatment (P=0.018, Figure 4F). Therefore, βE2 protection on primary cultured SD rat retinal cells was associated with transient Ca^2+^ influx gated by L-VGCC. 

### 3.5: βE2 pretreatment protected primary cultured SD rat retinal cells from H_2_O_2_-induced apoptosis by activating the PI3K pathway and then transiently up-regulating the [Ca^2+^]_i_

**Figure 5:**
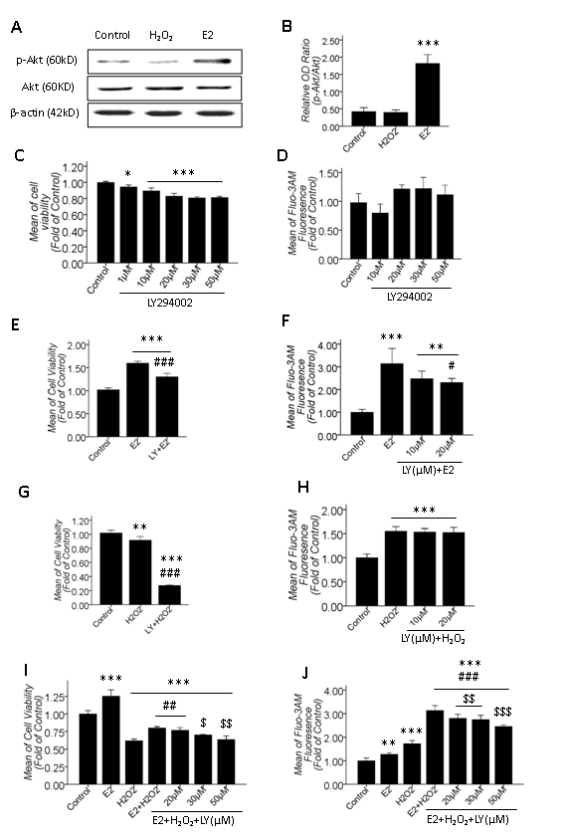
The effect of the PI3K inhibitor LY294002 (LY) on the cell viability and the [Ca^2+^]_i_ of primary cultured SD rat retinal cells in H_2_O_2_ injury and βE2 protection. A: Western blot results of the activation of the PI3K/Akt pathway after βE2 treatment for 0.5 hrs; B: Quantitative data of A; C, E, G, and I: Cell viability quantitative data; D, F, H, and J: [Ca^2+^]_i_ quantitative data; C and D: The effects of LY treatments for 24 hrs and 0.5 hrs on the cell viability and the resting [Ca^2+^]_i_; E and F: The inhibitory effect of LY pretreatment for 0.5 hrs on the increased cell viability and [Ca^2+^]_i_ induced by 10 μM βE2 treatment for 0.5 hrs (10 μM LY in E, 10 μM and 20 μM LY in F); G and H: The effect of LY pretreatment for 0.5 hrs on the decreased cell viability and increased [Ca^2+^]_i_ induced by 100 μM H_2_O_2_ treatment for 2 hrs (10 μM LY in G, 10 μM and 20 μM LY in H); I and J: The dose-dependent attenuating impact of 20-50 μM LY pretreatment for 0.5 hrs on the βE2 retinal protective role against H_2_O_2_ injury, which is associated with the dose-dependent attenuation of the increased [Ca^2+^]_i_ (Protocol of drug application: LY for 0.5 hrs, E2 for 0.5 hrs and H_2_O_2_ for 2 hrs). Values shown are the Mean ±SD. *represents P<0.05, **represents P<0.01 and ***represents P<0.001 compared with the control group; # represents P<0.05, ## represents P<0.01 and ### represents P<0.001 compared with the βE2 (E, F) or H_2_O_2_ (G, I, J) application groups; ＄ represents P＜0.05, ＄＄ represents P＜0.01 and ＄＄＄ represents P＜0.001 compared with the βE2 and H_2_O_2_ co-application group by one-way ANOVA statistical analysis. (B: n indicates 3 independent replicates; C, E, G, I: n indicates 3 independent replicates with 4 samples per condition per experiment; D, F, H, J: n indicates 3 independent replicates with 5 samples per condition per experiment.).

βE2 plays a protective role in the retina via the PI3K/Akt pathway [28]. Our results showed that βE2 protected primary cultured SD rat retinal cells from H_2_O_2_ injury, which was associated with a transient [Ca^2+^]_i_ increase (Figure 2). Therefore, we hypothesized that βE2 plays a protective role in our study model by activating the PI3K pathway and then transiently increasing [Ca^2+^]_i_. To test this hypothesis, we performed the following experiments using the PI3K inhibitor LY294002. First, we confirmed that 10 μM βE2 treatment for 0.5 hrs up-regulated the p-Akt level via Western blotting (Figure 5A, B). Second, we measured the effects of LY294002 on the cell viability and the [Ca^2+^]_i_ of the retinal cells and found that 1-50 μM LY294002 treatment for 24 hrs dose-dependently decreased the cell viability (Figure 5C), but treatment for 0.5 hrs had no effect on the resting [Ca^2+^]_i_ (Figure 5D). Third, we detected the inhibitory effects of LY294002 on the alteration of [Ca^2+^]_i_ and cell viability due to 10 μM βE2 treatment for 0.5 hrs or 100 μM H_2_O_2_ treatment for 2 hrs. Results showed that pretreatment for 0.5 hrs with 10 μM or 20 μM LY294002 significantly attenuated the increased cell viability and [Ca^2+^]_i_ due to βE2 (Figure 5E, F). However, 10 μM LY294002 did not reverse the cell viability decrease induced by H_2_O_2_ but instead promoted the decrease in cell viability (Figure 5G). In addition, both 10 μM and 20 μM LY294002 had no effect on the [Ca^2+^]_i_ increase induced by H_2_O_2_ (Figure 5H). PI3K was involved in the βE2-induced increase of [Ca^2+^]_i_ and cell viability but was not involved in the H_2_O_2_-induced [Ca^2+^]_i_ increase and cell viability decrease. Fourth, we verified that PI3K-mediated βE2 protection against H_2_O_2_ injury was associated with transiently up-regulating [Ca^2+^]_i_. As shown in Figures 5I and J, 20-50 μM LY294002 dose-dependently attenuated the βE2-mediated protective effect against H_2_O_2_ injury and dose-dependently restored the increased [Ca^2+^]_i_ induced by co-treatment with 10 μM βE2 for 0.5 hrs and 100 μM H_2_O_2_ for 2 hrs. 

**Figure 6:**
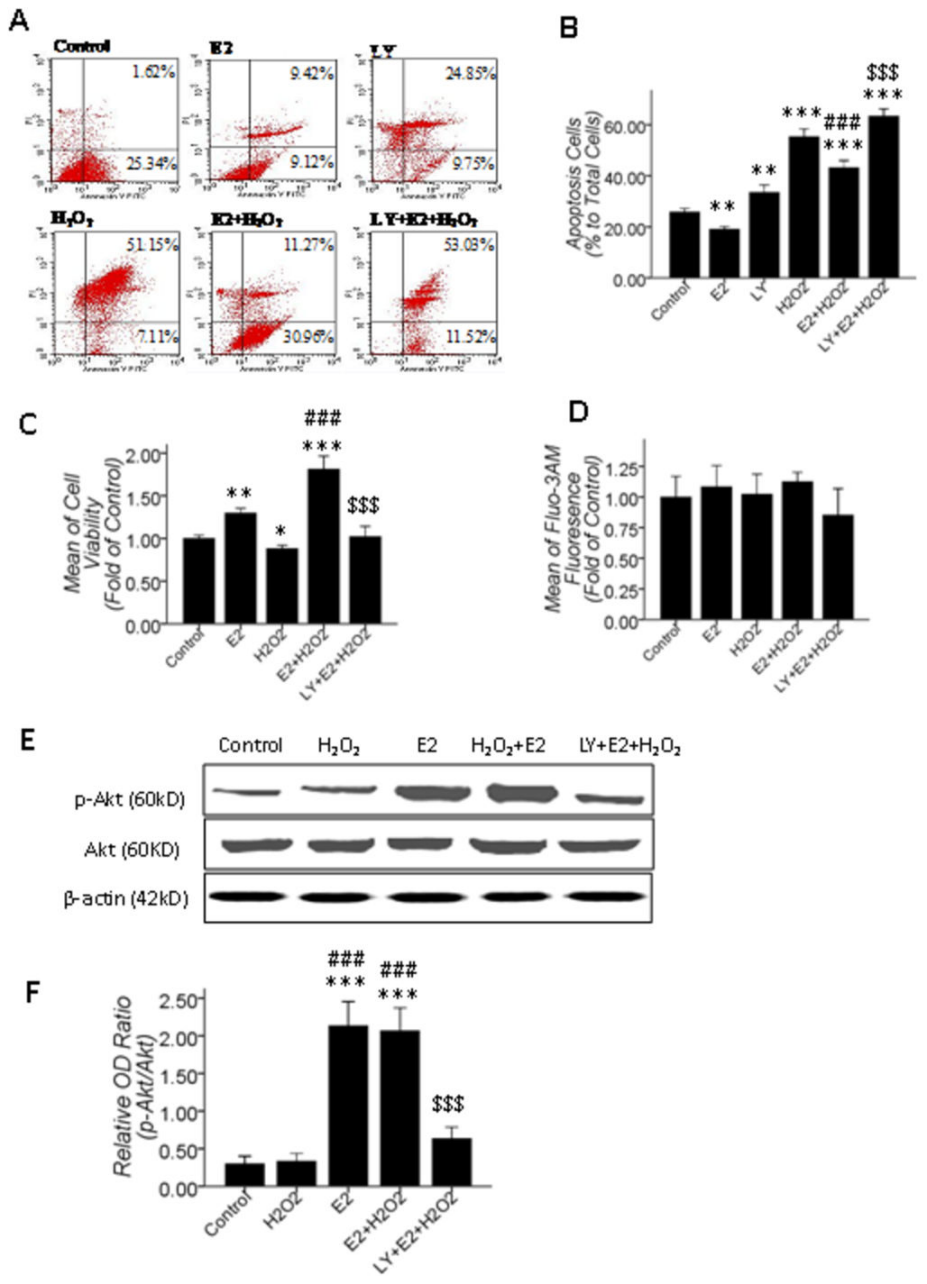
10 μM βE2 pretreatment for 0.5 hrs protected primary cultured SD rat retinal cells from apoptosis induced by 100 μM H_2_O_2_ treatment for 24 hrs. The PI3K/Akt pathway mediated this process, but the alteration in [Ca^2+^]_i_ was undetectable. A: The Annexin V/Propidium Iodide staining apoptosis assay; B: Quantitative data of A; C and D: Cell viability and [Ca^2+^]_i_ quantitative data; 10 μM βE2 pretreatment for 0.5 hrs significantly restored the decrease in cell viability and apoptosis, which was significantly inhibited by 10 μM LY (B, C), but the [Ca^2+^]_i_ was not significantly altered in all treated groups (D); E: Western blot results, 10 μM βE2 pretreatment for 0.5 hrs promoted p-Akt level, which was inhibited by 10 μM LY pretreatment for 0.5 hrs before βE2 and H_2_O_2_ co-treatment. F: Quantitative data of E. Values shown are the Mean ±SD. *represents P<0.05, **represents P<0.01 and ***represents P<0.001 compared with the control group by the T-test or one-way ANOVA statistical analysis; ### represents P<0.001 compared with the H_2_O_2_ application group by one-way ANOVA statistical analysis; $$$ represents P<0.001 compared with the βE2 and H_2_O_2_ co-application group by one-way ANOVA statistical analysis. (B, C, D: n indicates 3 independent replicates with 4 samples per condition per experiment; F: n indicates 3 independent replicates.).

Based on the results of cell viability and apoptosis assay in Figure 1D and H, 100 μM H_2_O_2_ treatment for 2 hrs led promoted retinal cell injury but not apoptosis. Therefore, we tested the role of βE2 in anti-apoptosis induced by 100 μM H_2_O_2_ for 24 hrs and the inhibitory effect of LY294002. In this experiment, we assayed the cell viability by the MTT assay and apoptosis by Annexin V/Propidium Iodide staining, and meanwhile, [Ca^2+^]_i_ measurements and Western blotting were performed. The results showed that 10 μM βE2 pretreatment for 0.5 hrs effectively protected the retinal cells from injury and apoptosis induced by 100 μM H_2_O_2_-mediated stressing for 24 hrs. Moreover, application of 10 μM LY294002 for 0.5 hrs before βE2 treatment significantly inhibited the βE2-mediated retinal protection against the H_2_O_2_-induced cell viability decrease and apoptosis (Figure 6A-C). Nevertheless, the [Ca^2+^]_i_ showed no alteration in all treated groups compared to the control group (Figure 6D), which further implicated that the βE2-induced increase in the [Ca^2+^]_i_ is an instantaneous event and that the [Ca^2+^]_i_ overload induced by H_2_O_2_ occurred during the early stage of apoptosis but did not occur at the later stages of apoptosis. Western blot results also showed that 10 μM βE2 pretreatment for 0.5 hrs markedly activated the PI3K/Akt pathway, which was significantly inhibited by 10 μM LY294002 (Figure 6E, F).

Briefly, the PI3K pathway mediated the βE2-induced [Ca^2+^]_i_ increase but did not mediate the H_2_O_2_-induced [Ca^2+^]_i_ increase. Pretreatment with 10 μM βE2 for 0.5 hrs protected primary cultured SD rat retinal cells from injury and apoptosis induced by H_2_O_2_ by activating the PI3K pathway, and then transiently up-regulated the [Ca^2+^]_i_, which was detectable at 2 h but not at 24 h after H_2_O_2_-induced stress.

## Discussion and Conclusion

[Ca^2+^]_i_ plays an important role in regulating most cellular processes and it is regulated by complex mechanisms. While brief elevations in [Ca^2+^]_i_ are required to control membrane excitability and to modulate essential processes, chronic elevations in [Ca^2+^]_i_ trigger toxic signaling cascades that lead to cell death [6,33–35]. Nevertheless, the selection of Ca^2+^ indicator and method of [Ca^2+^]_i_ measurement are very important as well as. They will affect the result of [Ca^2+^]_i_ measurement. Fluo-3 AM ester is a membrane-permeating form of fluo-3. It can passively diffuse across cell membranes and can be loaded into most of cells. Fluo-3 AM itself does not respond to Ca^2+^. However, once inside the cells, it is hydrolyzed to fluo-3 and can bind to Ca^2+^. Fluo-3 is one of the most suitable fluorescent Ca^2+^ indicators for ﬂow cytometry. It is a good probe because of its high sensitivity, but a few limited cells can be loaded directly with Ca^2+^ indicators [36]. Consequently, it is feasible and reasonable that we detected the [Ca^2+^]_i_ by FACS using Fluo-3 AM. The fluorescence of Fluo-3 AM precisely represents the actual [Ca^2+^]_i_. 

Recent evidence indicates that [Ca^2+^]_i_ is abnormal in many degenerative disorders in CNS. A number of studies suggest that alterations in [Ca^2+^]_i_ may result in cell apoptosis [37], which supports the relevance of [Ca^2+^]_i_ in the mechanisms leading to apoptosis. Several studies show that exposure to H_2_O_2_ induces the apoptosis of cultured neurons, which is mediated by increasing the [Ca^2+^]_i_. Several channels have been proposed to be involved in the H_2_O_2_-mediated [Ca^2+^]_i_ increase, including the N-methyl-D-aspartate (NMDA) receptor, the a-amino-3-hydroxy-5-methyl-4-isoxa-zole propionic acid (AMPA) receptor and VGCC [38–40]. The Transient Receptor Potential (TRP) protein superfamily is a group of voltage-independent Ca^2+^-permeable cation channels expressed in mammalian cells and consists of six subfamilies: TRPC, TRPV, TRPM, TRPA, TRPP, and TRPML [41,42]. Recent evidence suggests that Ca^2+^ inﬂux through TRP channels is an important mechanism through which oxidative stress mediates cell death and TRPC, and TRPM subfamily members are also activated by oxidative stress [42]. In our present study, we found that Ca^2+^ plays a substantial role in H_2_O_2_-induced apoptosis, and the [Ca^2+^]_i_ increase occurs at the early stage of apoptosis but not during the later stages of this process. Moreover, the increased [Ca^2+^]_i_ induced by H_2_O_2_ is partially caused by extracellular stores.

As for the mechanisms involved in βE2 retinal protection in our model, we speculated that βE2 resisted H_2_O_2_ stress by weakening the increased [Ca^2+^]_i_ due to H_2_O_2_. Inconsistent with our hypothesis, we found that 10 μM βE2 played a protective role by immediately sharpening but not restoring the increased [Ca^2+^]_i_ induced by H_2_O_2_. Furthermore, up to 2-5 mM doses of EGTA significantly attenuated the sharpening effect of βE2, indicating that this effect may be caused by a large Ca^2+^ transient influx. Many studies have proposed that L-VGCC plays an important role in the protective process in CNS, including retina [20–22,43]. In addition, several studies have indicated that the release of Ca^2+^ from the ER through the inositol 1, 4, 5-trisphosphate receptors (IP3Rs) is essential for cell survival and neuroprotection [44–46]. The members of the TRPM and TRPC subfamilies also play important roles in cell survival [47–50]. βE2 has been shown to be involved in the regulation of Ca^2+^ inﬂux via the TRPV5 channels [51], and preconditioned cells with a relatively low level of Ca^2+^ before an excitotoxic insult experienced neuroprotection in retinal ganglion cells [52]. Therefore, we hypothesized that βE2 increased the [Ca^2+^]_i_ through one or more relevant Ca^2+^ channels and signaling pathways. Excitedly, we discovered that the retinal protective role of βE2 through potentiating Ca^2+^ influx is controlled by L-VGCC and mediated by PI3K pathway.

Perplexedly, the results in our present study showed that both H_2_O_2_ injury and βE2 protection are mediated by increasing the [Ca^2+^]_i_ sourced from extracellular Ca^2+^ influx. These findings can be explained by the following ideas. First, Ca^2+^ exerts a biphasic effect on cellular growth, and a modest increase in [Ca^2+^]_i_ promotes cell proliferation, whereas relatively high [Ca^2+^]_i_ leads to increased mitochondrial Ca^2+^ and accounts for the release of pro-apoptotic factors resulting in cell death [8,9]. Second, a short increase in [Ca^2+^]_i_ is tolerated and may be needed to modulate biological functions, but the sustained increase in [Ca^2+^]_i_ leads to various degrees of cell damage until cell death. Third, under the two treatment conditions, the increased [Ca^2+^]_i_ may be due to different channels, and Ca^2+^ inﬂux through different routes may perform different biological functions [53]. For example, equally high Ca^2+^ loads are toxic when entering via the NMDA channels but not when entering via the VGCC [54]. Our present results showed that 2-12 hrs of a sustained [Ca^2+^]_i_ increase induced by H_2_O_2_ is harmful, but a transient [Ca^2+^]_i_ increase induced by βE2 for only 0.5 hrs is protective. Furthermore, the favorable [Ca^2+^]_i_ increase due to βE2 was gated by L-VGCC and was mediated by the PI3K pathway, but the harmful [Ca^2+^]_i_ increase caused by H_2_O_2_ was not gated by L-VGCC or mediated by the PI3K pathway. 

The majority of the results in this study are easily interpreted; nevertheless, several results are difficult to understand. For example, EGTA attenuated the increase of [Ca^2+^]_i_ induced by the 100 μM H_2_O_2_-induced injury (Figure 3E and F) but did not attenuate and inversely aggravated the decrease in cell viability (Figure 3D), which is most likely because extracellular Ca^2+^ is necessary for cell growth and chelating the extracellular Ca^2+^ leads to a decrease in cell viability. In our present study, we chelated the extracellular Ca^2+^, but we did not chelate the increased intracellular Ca^2+^, and we did not specifically block the channels controlling the extracellular Ca^2+^ influx due to the H_2_O_2_ injury. Further specific chelating and blocking experiments are being performed. Surprisingly, 20 μM nifedipine treatment for 0.5-1 hr increased the [Ca^2+^]_i_ significantly (Figure 4B); however, it was the reactively impermanent [Ca^2+^]_i_ increase. In this phenomenon, [Ca^2+^]_i_ may have reactively increased through other channels when L-VGCC was specifically blocked due to Ca^2+^ homeostasis at resting condition. After 0.5-1 hr of an impermanent [Ca^2+^]_i_ increase, the nifedipine developed its innate effect. However, this finding is novel and needs to be further investigated.

In summary, 100 μM H_2_O_2_-induced stress led to primary cultured SD rat retinal cell injury and apoptosis; however, 10 μM βE2 played a protective role on retinal cells. Both completely different roles were mediated by increasing the [Ca^2+^]_i_, which occurred at the early stage of 100 μM H_2_O_2_-induced apoptosis and 10 μM βE2 treatment for 0.5 hrs. Furthermore, the increase in [Ca^2+^]_i_ under completely opposite conditions were partially due to extracellular Ca^2+^ stores. Meaningfully, the transient [Ca^2+^]_i_ increase induced by βE2 was gated by L-VGCC, and the PI3K pathway was found to be involved but was not found to be involved in the H_2_O_2_-induced [Ca^2+^]_i_ increase. This finding may be due to different sources of Ca^2+^ through different channels activating pro-apoptotic or pro-survival pathways, thus performing the injury or the protective roles. Our present findings are very important for understanding the mechanism of retina degeneration and the search for preventative treatment targets. The detailed mechanisms and downstream signaling pathways of Ca^2+^ are mostly unknown; therefore, it is important to direct future efforts towards the mechanisms and pathways of βE2-mediated anti-apoptosis through regulating [Ca^2+^]_i_ and the downstream signals of Ca^2+^. 

The data from our present study were based on a primary mixed cell culture of retinal cell population. The in vitro model is widely used for studying the pathogenesis of diseases. The primary retinal cell culture began in the late 1950’s. Today, it is routinely used for studies and remains the most widely-used form of retinal cell culture [55]. Moreover, mixed primary culture of retinal cell population includes various retinal cells and may better represent the in vivo condition than a cell line. Besides, H_2_O_2_ triggers apoptosis and becomes a well-established in vitro model for studying the pathology of oxidative stress in degenerative disorders of CNS such as AMD, which is relative to the producing of reactive oxygen species (ROS) [11,13]. Therefore, the model of H_2_O_2_-induced apoptosis of primary cultured retinal cells represents the pathogenesis of multiple retinal degenerative diseases. Certainly, in our future studies, we will do some research using in vivo model to obtain results that are more closely applicable to in vivo conditions.
